# Experimental validation of convection-diffusion discretisation scheme employed for computational modelling of biological mass transport

**DOI:** 10.1186/1475-925X-9-34

**Published:** 2010-07-19

**Authors:** Gráinne T Carroll, Paul D Devereux, David N Ku, Timothy M McGloughlin, Michael T Walsh

**Affiliations:** 1 Centre for Applied Biomedical Engineering Research (CABER), Department of Mechanical and Aeronautical Engineering, and the Materials and Surface Science Institute, University of Limerick, Limerick, Ireland; 2 G. W. Woodruff School of Mechanical Engineering, Georgia Institute of Technology, Atlanta, Georgia 30332-0405 USA

## Abstract

**Background:**

The finite volume solver Fluent (Lebanon, NH, USA) is a computational fluid dynamics software employed to analyse biological mass-transport in the vasculature. A principal consideration for computational modelling of blood-side mass-transport is convection-diffusion discretisation scheme selection. Due to numerous discretisation schemes available when developing a mass-transport numerical model, the results obtained should either be validated against benchmark theoretical solutions or experimentally obtained results.

**Methods:**

An idealised aneurysm model was selected for the experimental and computational mass-transport analysis of species concentration due to its well-defined recirculation region within the aneurysmal sac, allowing species concentration to vary slowly with time. The experimental results were obtained from fluid samples extracted from a glass aneurysm model, using the direct spectrophometric concentration measurement technique. The computational analysis was conducted using the four convection-diffusion discretisation schemes available to the Fluent user, including the First-Order Upwind, the Power Law, the Second-Order Upwind and the Quadratic Upstream Interpolation for Convective Kinetics (QUICK) schemes. The fluid has a diffusivity of 3.125 × 10^-10 ^m^2^/s in water, resulting in a Peclet number of 2,560,000, indicating strongly convection-dominated flow.

**Results:**

The discretisation scheme applied to the solution of the convection-diffusion equation, for blood-side mass-transport within the vasculature, has a significant influence on the resultant species concentration field. The First-Order Upwind and the Power Law schemes produce similar results. The Second-Order Upwind and QUICK schemes also correlate well but differ considerably from the concentration contour plots of the First-Order Upwind and Power Law schemes. The computational results were then compared to the experimental findings. An average error of 140% and 116% was demonstrated between the experimental results and those obtained from the First-Order Upwind and Power Law schemes, respectively. However, both the Second-Order upwind and QUICK schemes accurately predict species concentration under high Peclet number, convection-dominated flow conditions.

**Conclusion:**

Convection-diffusion discretisation scheme selection has a strong influence on resultant species concentration fields, as determined by CFD. Furthermore, either the Second-Order or QUICK discretisation schemes should be implemented when numerically modelling convection-dominated mass-transport conditions. Finally, care should be taken not to utilize computationally inexpensive discretisation schemes at the cost of accuracy in resultant species concentration.

## Background

Biological mass transport analyses examine species transport within the vasculature. More specifically, blood side mass transport (BSMT) investigations attempt to identify regions in the vasculature where the mass transport (MT) of blood borne elements to the endothelium is disturbed by the local fluid mechanics of blood. Computational fluid dynamics (CFD) is a numerical technique used to solve both fluid flow and mass transport problems. The most common numerical techniques are the finite volume method (FVM) and the finite element method (FEM). The FVM is typically implemented using commercially available software packages such as Fluent 6.1 (Lebanon, NH, USA) [1-5], CFX (Ontario, Canada) [6,7] or CFDS-Flow3D [8], where as the FEM is typically applied via solvers that are developed "in house" [9-13], although commercial FEM solvers are also available, such as FIDAP (Fluent Inc., Lebanon, NH, USA) and ADINA (Watertown, MA, USA) [14]. The current study utilises the FV solver, Fluent 6.1 (Fluent *Inc*., Lebanon, NH, USA), for fluid flow and mass transport analysis within an idealised aneurysm geometry.

Previous finite volume analyses of BSMT include research carried out by Lei *et al*., 1996, Ma *et al*., 1997 and Devereux *et al*., 2005[1,3,8]. This research has examined the influence of geometry and local haemodynamics on MT disturbances of various species and their combined role in the development of atherosclerosis and intimal hyperplasia. However, within this body of research details of either theoretical or experimental validation of the computational results are limited, with no justification of the choice of convection-diffusion discretisation schemes employed. In 1996, Lutostansky compared a finite volume blended second order central differencing and upwinding scheme to the streamline upwind/Petrov-Galerkin (SU/PG) FE method and to experimentally obtained results [15]. However, this earlier finite volume convection-diffusion discretisation scheme may be prone to numerical instabilities and as a result has been discontinued by Fluent (Personal communication, Fluent Inc.). To this end, the focus of the present study is the experimental validation of the FVM for blood-side (convection dominated) MT, specifically the choice of convection-diffusion discretisation scheme within Fluent 6.1.

The FVM consists of three distinct steps. Firstly, the discretisation process includes the formal integration of the governing equations over each control volume in the solution domain, yielding a discrete set of equations that conserve each quantity on a control volume basis. These integral equations are then converted into a system of algebraic equations via the substitution of a variety of finite-difference-type approximations for the convection, diffusion and source terms in the integrated equation and finally, solution of the algebraic equations by an iterative method. In Fluent a number of upwinding schemes for solution discretisation are available to the user, namely the First-Order Upwind Scheme, the Power Law scheme, the Second-Order Upwind Scheme and the Quadratic Upstream Interpolation for Convective Kinetics (QUICK) Scheme.

Implicit FV methods provide a relatively quick and efficient solution to multi-dimensional simulations involving fluid flow and mass transport. However, due to the numerous solution options available to the user when developing a numerical model, there is a basic requirement that any computational results obtained should either be validated against benchmark theoretical solutions or against experimentally obtained results. When computationally modelling BSMT a principal consideration is the choice of convection-diffusion discretisation scheme employed due to the possibility of non-physical instabilities and numerical diffusion. Thus, experimental validation of the solution discretisation scheme is required to ensure the accuracy of the results obtained. This study presents the computational MT results within a standard idealised aneurysm model for all four discretisation schemes available in the FV solver Fluent. The numerical findings are compared to experimental results, obtained using the direct spectrophometric concentration measurement technique, to identify the most accurate convection-diffusion discretisation scheme for the computational modelling of BSMT.

## Methods

### Experimental and Computational Geometry

**Figure 1:**
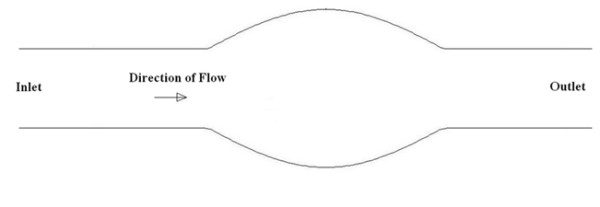
**Idealised axisymmetric aneurysm model utilised for both computational and experimental analysis**. Maximum aneurysm diameter is twice inlet/outlet diameter of 25.1 mm.

The idealised aneurysm geometry, as seen in figure 1, consisted of a 25.1 mm diameter inlet and outlet with the aneurysm extending to a maximum diameter of 50.2 mm. The distance from inlet of the aneurysm to its outlet was 75.3 mm. An axisymmetric aneurysm model was chosen for the experimental and computational analysis as it has a well-defined recirculation region within the aneurysmal sac, within which the species concentration varies slowly with time.

### Experimental Fluid Flow Boundary Conditions

**Figure 2:**
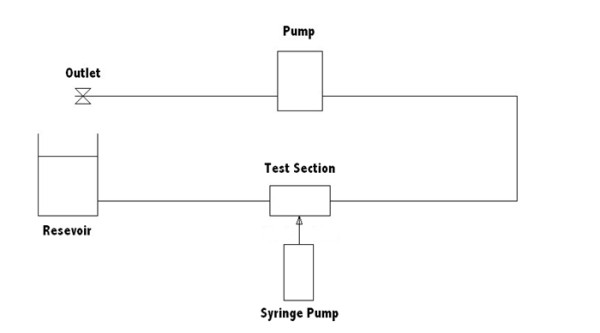
**Schematic of experimental flow system**. Test section refers to idealised aneurysm model.

A constant volumetric flow rate of 3.94 × 10^-6 ^m^3^/s was applied via a centrifugal pump (MCP-Z, Ismatec Inc.) resulting in a Reynolds number of 800. The resulting Peclet number for this system is 2,560,000 indicating strongly convection-dominated flow. A schematic of the experimental flow system is presented in figure 2.

#### Species of Interest

The food dye FD&C Blue #1 was used as the transported species. The diffusivity of the dye was calculated using the Stokes-Einstein equation. Assuming a spherical molecule, the characteristic radius of the molecule can be calculated using the molecular weight of the dye. The Stokes-Einstein diffusivity of a molecule of this size in water is 3.125 × 10^-10 ^m^2^/s, resulting in a Schmidt number of 3200, which is in the range of blood borne species such as oxygen free radicals [13,16].

### Experimental Methodology: The Direct Spectrophometric Concentration Measurement Technique

**Figure 3:**
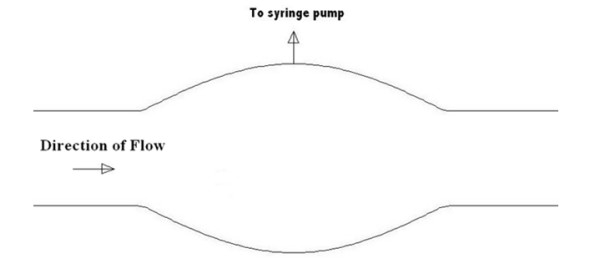
**Idealised axisymmetric aneurysm model utilised for both computational and experimental analysis**. Volume fluid extraction was conducted using syringe pump.

The technique used in this analysis involves the extraction of small volumes of fluid from the test section using a syringe pump, figure 3, at numerous time-points over the course of the experiment. As the concentration field in the test section is time dependent until equilibrium is reached, the concentration values in each extracted volume would be expected to vary over the time course of the experiment. This technique was developed by Lutostansky (1996) [15] and has been presented in previous publications [17].

Withdrawal of the species sample from the geometry is achieved via a small diameter hole (<1 mm) in the experimental glass model of the idealised aneurysm. This hole was positioned at the location of maximum aneurysm diameter (figure 3). A small-bore syringe needle is placed flush to the inner surface of the glass model. By using a syringe pump (Harvard Apparatus, Holliston, MA, USA) it is possible to accurately extract small volumes of fluid from the test section. During the experiments carried out in this study, a volume of 0.8 ml was withdrawn over the course of one minute. The experiment was 7 minutes in duration and 0.8 ml of fluid was extracted every second minute starting at time equal to zero. Time zero was determined as the time at which the food dye entered the aneurysm section of the experimental model.

#### Experimental Measurement of Species Concentration

A spectrophotometer (SpectraMax Plus^384^, Molecular Device) was used to determine the concentration of food dye (FD&C Blue #1) in each sample volume at a wavelength of 630 nm. Both inlet concentration and zero concentration (water) were also determined and the results are presented in terms of a normalised concentration value.

### Computational Fluid Dynamics

The computational model was built, subdivided and meshed in Gambit 2.1 (*Fluent Inc. Lebanon, NH, USA)*. A structured quadrahedral grid was created for the idealised aneurysm model. The computational grid employed in this study has an equiangle skew value of less than 0.7 and an aspect ratio magnitude < = 5. Grid independence was established for Sherwood number, and error was specified to within ± 2%. The final grid size was identical for each method, consisting of 280,000 quadrahedral elements in the 2-D axisymmetric model.

The finite volume solver Fluent 6.1 was used to solve the two-dimensional, incompressible, Navier-Stokes equations. Discretisation of the governing momentum equations at each control volume was achieved by the Quadratic Upwind Interpolation for Convective Kinetics (QUICK) scheme, employing a three-point upstream-weighted quadratic interpolation for the cell face values. The Pressure Implicit with Splitting of Operators (PISO) pressure-velocity coupling algorithm was employed for this analysis, due to the time-dependent nature of the concentration profile. A convergence criterion of 1 × 10^-3 ^was defined for the residuals of the continuity, momentum, and species transport equations [1,18].

### Convection-Diffusion Discretisation

The process of discretisation is presented here and is most easily illustrated by considering the steady-state conservation equation for the transport of a scalar quantity, ϕ:

(1) $$\int \rho \phi \vec{v}.d\vec{A}=\int \Gamma_{\phi }\nabla \phi .d\vec{A}+\int_{V}S_{\phi }dV$$

where *ρ *equals the fluid density, $\vec{v}$ is the velocity vector, Γ_ϕ _is the diffusion co-efficient of *ϕ *, and *S_ϕ _*is the source of *ϕ *per unit volume. This equation is applied to each control volume in the computational domain. Discretisation of equation 1 on any given cell yields:

(2) $$\sum_{f}^{N_{faces}}\rho_{f}\vec{v}\phi_{f}.\vec{A_{f}}=\sum_{f}^{N_{faces}}\Gamma_{\phi }{\left(\nabla \phi \right)}_{n}.\vec{A_{f}}+S_{\phi }V$$

and this is the general form of the equations solved by Fluent.

Discrete values of *ϕ *are stored at cell centres and are used to solve the diffusion term in equation 2. However, the convection term requires face values of the scalar quantity, *ϕ_f_*, which must be interpolated from the cell centre values. This is achieved by implementing an upwinding scheme. Upwinding involves deriving cell face values from quantities in the cell upstream, or "upwind", relative to the direction of the normal velocity, *ν_n_*, in equation 2. The upwinding schemes for solution discretisation available in Fluent 6.1 are First-Order Upwind, Second-Order Upwind, Power Law and QUICK. These four upwind schemes will now be introduced but further, more detailed, information is provided by Versteeg and Malalasekera, 1995 [19,20].

#### First-Order Upwind Scheme

Quantities derived from this upwind scheme are determined by assuming that the cell-centre values of any field variable represent a cell average value and hold throughout the entire cell, resulting in face values that are identical to cell-centre values. Thus, by selecting this upwind scheme, the face value, *ϕ_f_*, is equal to the cell-centre value of, *ϕ *in the upstream cell.

#### Power Law Scheme

This discretisation scheme involves the interpolation of, *ϕ_f _*by using the exact solution to the one-dimensional convection-diffusion equation:

(3) $$\frac{\partial }{\partial x}\left(\rho u\phi \right)=\frac{\partial }{\partial x}\Gamma \frac{\partial \phi }{\partial x}$$

where Γ and *ρu *are constants across the interval ∂*x*. Integration of equation 3 yields the following equation that describes how *ϕ *varies with *x*:

(4) $$\frac{\phi \left(x\right)-\phi_{0}}{\phi_{L}-\phi_{0}}=\frac{e^{\left(Pe\frac{x}{L}\right)}-1}{e^{\left(Pe\right)}-1}$$

where Pe is the Peclet number.

**Figure 4:**
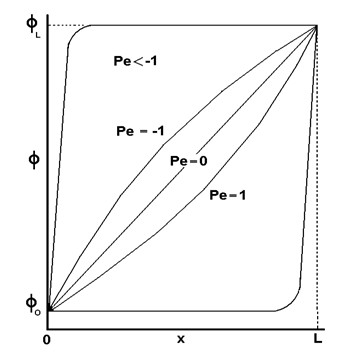
**Variation of *ϕ *between *x *= 0 and *x *= L [Developed from Fluent, **[19]**]**.

The variation of the scalar quantity between *x *= 0 and *x *= L is depicted in figure 4. This figure illustrates that for large Pe values (convection dominated flow), the value of *ϕ *at = L/2 is approximately equal to the upstream value, an assumption that is identical to the First-Order Upwind scheme described above.

#### Second-Order Upwind Scheme

When second-order accuracy is desired, a Taylor series expansion is used to calculate *ϕ_f _*via the following equation:

(5) $$\phi_{f}=\phi +\nabla \phi .\Delta \vec{s}$$

where *ϕ *and ∇*ϕ *are the upstream cell centred value and its gradient respectively, and $\Delta \vec{s}$ is the displacement vector from the upstream cell centroid to the face centroid. The gradient of *ϕ *needs to be determined in order to solve equation 5. This is achieved using the divergence theorem, which in discrete form is written as

(6) $$\nabla \phi =\frac{1}{V}\sum_{f}^{N_{faces}}{\tilde{\phi }}_{f}\vec{A}$$

In equation 6, the face values ${\tilde{\phi }}_{f}$ are computed by averaging *ϕ *from the two cells adjacent to the face.

#### QUICK Scheme

The quadratic upstream interpolation for convective kinetics (QUICK) scheme is used to compute a higher-order value of the convected variable *ϕ *at a face and uses a three-point upstream-weighted quadratic interpolation.

**Figure 5:**
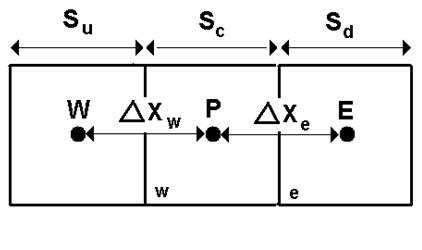
**One-Dimensional control volume [Developed from Fluent, **[19]**]**.

For face *e *in figure 5 and assuming the flow is from left to right, the face value *ϕ_e _*can be written as:

(7) $$\phi_{e}=\psi \left[\frac{S_{d}}{S_{c}+S_{d}}\phi_{p}+\frac{S_{c}}{S_{c}+S_{d}}\phi_{E}\right]+(1-\psi )\left[\frac{S_{u}+2S_{c}}{S_{u}+S_{c}}\phi_{p}-\frac{S_{c}}{S_{u}+S_{c}}\phi_{W}\right]$$

The traditional QUICK scheme is obtained by setting *Ψ *= 1/8 in equation 7.

### Boundary Conditions

#### Inlet Boundary Conditions

An identical volumetric flow rate to that applied in the experimental analysis was applied at the inlet of the computational model, resulting in a Reynolds number of 800 and a Peclet number of 2,560,000. Due to the time-dependent nature of the concentration profile until equilibrium is reached, an unsteady analysis was modelled implementing a time step size of 1 second. The bulk carrier fluid was assumed to be Newtonian, isothermal, and incompressible [21], with a density of 1000 kg/m3 and a dynamic viscosity of 0.001 Pas. A constant mass fraction of the species of interest was specified at the inlet.

#### Outlet Boundary Condition

An outflow boundary condition was applied to the outlet of the geometry, allowing the outflow values of pressure and velocity to be extrapolated from the interior of the computational mesh.

#### Symmetry Boundary Condition

By taking advantage of the symmetry of the experimental model, an axisymmetric boundary condition along the centre axis of the computational model was applied thus reducing computational effort.

#### Wall Boundary Condition

The model wall was assumed rigid with a no-slip boundary condition. To simulate the glass wall condition of the experimental model, the wall boundary was assumed to be an inert material with zero mass flux normal to the wall surface. Although this wall condition is not the physiologically consistent choice it can be considered physiologically relevant when the permeability of the arterial wall to a species is very low and the event is reaction limited.

### Computational Concentration Measurements

**Figure 6:**
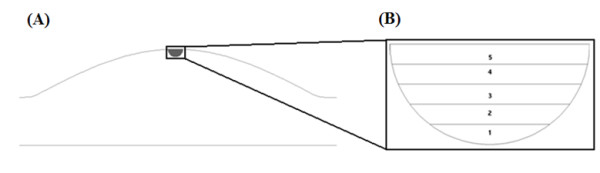
**(A) Illustration of experimental hemispherical cap of fluid extracted from idealised aneurysm**. (B) Sub-division of the idealised hemisphere for concentration quantification of computational models.

The idealisation that the volume of fluid extracted from the experimental geometry represents a hemisphere was assumed in the computational analysis (figure 6(A)). A hemisphere with a volume of 0.8 ml has a radius of 3.37 mm. To obtain the computational results this hemisphere was subdivided into 5 separate volumes of equal height as shown in figure 6(B). The location of the centroid of these five volumes was determined and the concentration value at each of these points was obtained. These values were weighted in accordance to the corresponding volume of fluid in which each centroid was located. A volume weighted average value for concentration was subsequently obtained. This method allowed for a direct comparison to be made between the computationally obtained results to those obtained experimentally.

Four separate computational models were completed with the only variation between the models being the discretisation scheme applied to the solution of the species convection-diffusion equation. The results obtained were compared to determine whether or not the upwinding scheme influenced the computational results. Furthermore, in an attempt to determine the influence of the discretisation scheme selection on the accuracy of the MT solution and to validate the computational analysis of the species convection-diffusion equation, the experimental results were compared to the results obtained from a CFD analysis of the aneurysm geometry.

## Results

**Figure 7:**
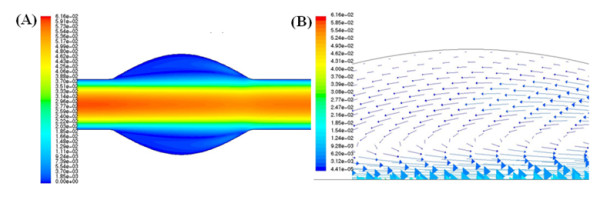
**Typical contours and vectors of velocity within the idealised aneurysmal sac**. (A) Velocity contours in computational aneurysm model (ms^-1^). (B) Velocity vectors within the recirculation region (ms^-1^).

An idealised aneurysm model was chosen for this study. It has a number of advantages, namely the recirculation region within an idealised aneurysm model is well defined. Furthermore, within this recirculation region, species concentration varies slowly with time, thus allowing for the withdrawal of small volumes of fluid during the course of the experimental part of the analysis. Typical contours of velocity magnitude within the geometry are illustrated in figure 7(A) and velocity vectors within the recirculation region are presented in figure 7(B).

**Figure 8:**
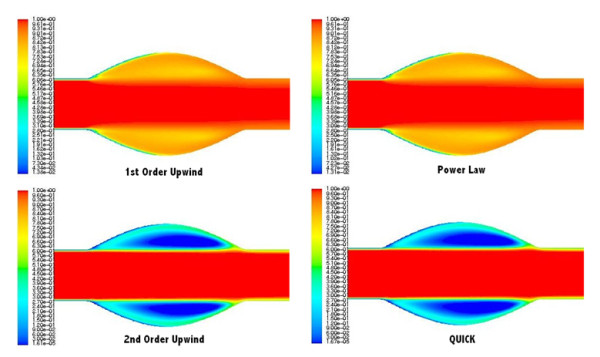
**Contours of normalised species concentration as determined by the four different convection-diffusion upwinding techniques, T = 4 mins**: First-Order Upwind Scheme, Power Law scheme, Second-Order Upwind Scheme and Quadratic Upstream Interpolation for Convective Kinetics (QUICK) Scheme.

The contours of species as determined by the four discretisation "upwinding" schemes examined computationally are presented in Figure 8. In each computational model presented, the contours of species are normalised by the inlet concentration and are representative of the concentration field 4 minutes after the inlet concentration has entered the test section.

It is evident from figure 8 that the choice of upwinding technique has a strong effect on the concentration field as determined by CFD. First-Order Upwind and the Power Law schemes produce similar results. The Second-Order Upwind and QUICK schemes also produce near identical results, illustrating well defined concentration gradients present perpendicular to the direction of flow within the aneurysmal sac, but differ strongly from the First-Order Upwind and Power Law concentration contour plots.

**Figure 9:**
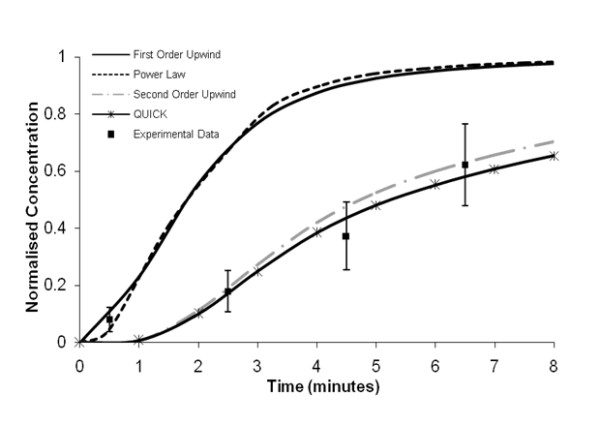
**Comparison between computational and experimental normalised species concentration results**. The experimental data presented is the average values of eight sets of experimental results.

Figure 9 quantitatively illustrates the variance introduced into the computational solution by the application of different discretisation schemes. The experimental data correlates well with both the Second-Order Upwind and QUICK discretisation schemes with an average percentage difference of 8% and 15%, respectively, indicating that either upwinding technique should be applied to the solution of the species convection-diffusion equation. The experimental data outlined in figure 9 is presented as the average values of eight sets of experimental results with error bars that represent a 95% confidence interval. The computational results obtained using the First-Order Upwind or Power Law discretisation scheme would over predict the normalised concentration within the idealised model by an approximate temporal average of 140% and 116%, respectively, when compared to the experimental results. At each individual time-point, an increase in the experimental data of two standard errors would still greatly under predict the computational results obtained using either a First Order Upwind or Power Law discretisation scheme. Potential reasons why such variations exist between the computational and experimental results shall now be discussed.

## Discussion

When computationally modelling species MT, a number of validation assessments are required to ensure accuracy and repeatability, including experimental validation of the discretisation scheme used to solve the convection-diffusion equation. The aim of this paper is to examine concurrently, for the first time, all convection-diffusion discretisation schemes available in Fluent and to identify which numerical scheme is the most appropriate for numerical modelling of high Peclet number MT processes.

Four convection-diffusion upwinding schemes are available to the FV Fluent user, namely the First-Order Upwind, Power Law, Second-Order Upwind and QUICK discretisation schemes. The First-Order Upwind technique assumes that the cell-centre value of any field variable represents the cell average value, producing face values that are equal to the cell-centre value. Similar to the First-Order Upwind technique, in the case of convection dominated MT, the Power Law scheme assumes that the value of the field viable (*Ø*) at L/2 is approximately equal to the upstream value. The Second-Order Upwind scheme utilises a Taylor series expansion to calculate the field variable (*Ø_f_*). It does not make the assumption that the cell-centre value is equal to the face value but instead computes the face value from the average of the two adjacent cells. The QUICK discretisation scheme is used to compute a higher-order face value of the convection variable *ϕ *and uses a three-point upstream-weighted quadratic interpolation.

This investigation initially demonstrated the influence of the upwinding technique employed for convection-diffusion discretisation on concentration fields within an idealised aneurysm model. The results were then compared to concentrations obtained experimentally from the direct spectrophometric concentration measurement technique. This experimental methodology is similar to that conducted by Lutostansky *et *al., 2003, who compared experimental results in a sudden expansion flow chamber to those obtained using an in house developed FE code and Fluent [22]. The findings indicated a positive correlation between the finite element, finite volume and experimental results. However, only one FV discretisation scheme was evaluated by Lutostansky *et al.*, 2003 [22]. In this study, four discretisation schemes were analysed and compared to experimental data. Thus, this research identifies the most suitable FV discretisation upwinding scheme to accurately predict the local mass transport behaviour and species concentration under convection dominated flow conditions for computational BSMT investigations as either the Second Order Upwind or QUICK schemes.

The choice of upwinding scheme for solution discretisation has a major influence on concentration gradients perpendicular to the direction of flow and time-dependent normalised concentration within the aneurismal sac of the idealised model. In agreement with theory, under convection dominated flow, the First-Order Upwind and the Power Law schemes produce near identical results. The results obtained from the Second-Order Upwind and QUICK schemes correlate well but were found to differ considerably from those of the First-Order Upwind and Power Law schemes.

Upon comparison of the experimental and computational concentration results, the inadequacy of both the First-Order Upwind and Power law discretisation schemes, with respect to the species convection-diffusion equation, to predict high Peclet number MT was clearly demonstrated. It was found that the Second-Order Upwind scheme or the QUICK scheme are the most suitable upwinding schemes for the computational modelling of convection dominated biological MT. It is well documented that the First-Order Upwind discretisation scheme is subject to non-physical instabilities and numerical diffusion [20,23]. Such false diffusion occurs when flow is not aligned with the grid lines of the computational mesh resulting in distributions of the transported properties that become smeared. Both the First-Order Upwind and Power Law schemes provide a similar solution. This is to be expected as the Power Law scheme reverts to the First-Order Upwind scheme in high Peclet number flows, as present in this study. Both the Second-Order Upwind and the QUICK schemes provide nearly identical results with well-defined sharp concentration gradients perpendicular to the direction of flow. This sharpness of the concentration gradients is due to the fact that both the Second-Order and QUICK discretisation schemes are not subject to false diffusion. The findings of this experimental analysis suggest that when modelling convection dominated species transport, a Second-Order Upwind or QUICK discretisation scheme must be implemented in order to obtain accurate convection dominated MT results.

Implicit finite volume methods (FVM) have become more popular in multi-dimensional flow simulations due to their inherent efficiency in terms of memory requirement and CPU time consumption [23]. Although computational time for MT analysis increases with utilisation of more complex convection-diffusion discretisation schemes, the significant increase in global computational cost due to the mesh requirements of lower order schemes indicates that overall solution accuracy and computational time is optimised using higher-order discretisation schemes. The large percentage errors evidenced when employing the First-Order and Power Law schemes, 140% and 116% respectively, highlight the importance of using higher order convection-diffusion discretisation schemes for high Peclet number MT processes.

## Conclusions

This study serves as an experimental validation of the FV numerical procedures utilised for vascular BSMT investigations. It was demonstrated that for convection dominated MT analyses the choice of convection-diffusion discretisation scheme has a strong influence on resultant species concentration fields. The First-Order Upwind and the Power Law schemes produce similar results but differ significantly from the concentration contours of the Second-Order Upwind and QUICK schemes, which also correlate well. Experimental validation of numerical BSMT analysis not only provides a level of confidence in the results obtained from Fluent but also serves as an indicator as to which discretisation scheme should be applied to the solution of the species convection-diffusion equation. A comparison between the computational findings and the experimentally obtained results suggests that the Second-Order or QUICK discretisation schemes should be implemented when numerically modelling convection-dominated MT conditions.

## Competing interests

The authors declare that they have no competing interests.

## Authors' contributions

GTC analysed the results and prepared the manuscript. PDD conducted the computational and experimental analysis, analysed the results, DNK supervised the experimental research, TMM supervised the study, revised the manuscript, MTW supervised the study, revised and gave the final approval of the manuscript. All authors read and approved the final manuscript.
